# RNA sequencing analyses reveal differentially expressed genes and pathways as Notch2 targets in B-cell lymphoma

**DOI:** 10.18632/oncotarget.27805

**Published:** 2020-12-01

**Authors:** Ashok Arasu, Pavithra Balakrishnan, Thirunavukkarasu Velusamy

**Affiliations:** ^1^Department of Biotechnology, School of Biotechnology and Genetic Engineering, Bharathiar University, Coimbatore, India

**Keywords:** SMZL, Notch2, RNA sequencing, PI3K/AKT, NF-kB

## Abstract

Splenic marginal zone lymphoma (SMZL) is a low grade, indolent B-cell neoplasm that comprises approximately 10% of all lymphoma. Notch2, a pivotal gene for marginal zone differentiation is found to be mutated in SMZL. Deregulated Notch2 signaling has been involved in tumorigenesis and also in B-cell malignancies. However the role of Notch2 and the downstream pathways that it influences for development of B-cell lymphoma remains unclear. In recent years, RNA sequencing (RNA-Seq) has become a functional and convincing technology for profiling gene expression and to discover new genes and transcripts that are involved in disease development in a single experiment. In the present study, using transcriptome sequencing approach, we have identified key genes and pathways that are probably the underlying cause in the development of B-cell lymphoma. We have identified a total of 15,083 differentially expressed genes (DEGs) and 1067 differentially expressed transcripts (DETs) between control and Notch2 knockdown B cells. Gene Ontology (GO) term enrichment and pathway analysis were applied for the identification of key genes and pathways involved in development of B-cell lymphoma. In addition, intermediate genes of top canonical pathways such as PI3K/AKT and NF-kB were found to be downregulated with Notch2 knockdown, indicating that these pathways could be the putative downstream effectors through which Notch2 mediates its oncogenic effects. Taken collectively, the identified crop of genes and pathways may be considered as targets for the treatment of B-cell lymphoma.

## INTRODUCTION

Marginal zone lymphomas (MZL’s) are group of indolent B-cell lymphomas that originate from the marginal zone of B-cell follicles. The World Health Organization (WHO) classified MZL into three types based on the site of origin and characteristics, splenic marginal zone lymphoma (SMZL), nodal marginal zone lymphoma and extranodal mucosa-associated lymphoid tissue (MALT) also known as MALT lymphoma [1, 2]. Splenic marginal zone lymphoma (SMZL) is a low grade B-cell non-Hodgkin’s lymphoma (NHL) and it is the most common primary malignancy of the spleen which accounts for ~10% of all lymphomas. It is an uncommon form of NHL that represents less than 1% of new cases [1, 3, 4]. Unlike the majority of other B-cell lymphomas, SMZL was not associated with recurrent chromosomal translocations or genetic mutations until recently. Notch2, a key regulator for marginal zone differentiation and homing of B cells to the splenic marginal zone were found to be most frequently mutated gene in SMZL [5–8]. Exome sequencing showed that high-frequency recurrent somatic mutations in the Notch2 pathway were present in 30% of tumors with mutations in the C-terminal PEST domain of Notch2 that controls its proteasomal degradation [7, 9, 10]. *Notch1* is mutated in only about 5% of SMZLs [9, 11] and mutations in *SPEN*, *DTX1*, and *MAML2* were also reported with a least percentage (5%) of SMZL cases [9]. Thus, *Notch2* mutations were found to be highly penetrant and specific for marginal zone lymphomas, when compared to other B-cell leukemias and lymphomas [7].

Aberrant Notch2 signaling and increased Notch2 expressions were observed in diverse human cancers including chronic lymphocytic leukemia (CLL) [12, 13], marginal zone lymphoma (MZL) [7, 9], breast cancer [14], non-small cell lung cancer [15], pancreatic cancer [16], hepatocellular carcinoma (HCC) [17], colorectal cancer [18], bladder cancer [19], medulloblastoma [20] and glioblastoma [21, 22] (Supplementary Figure 1). Emerging reports have established the oncogenic role of Notch2 signaling and its deregulation have been implicated in variety of cancers which makes Notch2 an excellent candidate for investigation [7, 9]. Hence, in recent years Notch2 has become an interesting target for pharmacological intervention.

A wide range of Notch2 mutations have been identified with relevance to different cancers, but the role of Notch2 and its downstream pathways in development of B-cell lymphoma has not been comprehensively studied to date. Available reports evident that, majority of cancers especially B-cell malignancies have also been associated with overexpression of Notch2 or Notch2 gain-of-function mutations that enhances Notch2 activity, causing tumorigenesis [23]. Considering the fact that Notch2 functions as key factor in mediating B cell malignancies, deeper studies are needed to pinpoint the contribution of Notch2 in the hierarchy of events driving B cell transformation [23, 24]. With the advancement of the next-generation sequencing technologies, RNA sequencing (RNA-Seq) has become a useful tool in defining the transcriptome of cells. The transcriptomics is essential for interpreting the functional elements of the genome and also for understanding development and disease [25].

The current study is the first of its kind, wherein comprehensive transcriptome analysis using RNA-Seq was performed in Notch2 depleted B-cell lymphoma cells. This study provides new insights on novel downstream targets and pathways that are modulated by Notch2 in B-cell lymphoma. Based on RNA-Seq technology, the integrated global gene expression and signaling pathway activities in B-cell lymphoma were measured after knockdown of Notch2. The study provides novel insights on differentially expressed genes at the transcriptome level and the putative tumour promoting molecular pathways that are influenced in response to Notch2 knockdown. Transcriptome analysis thus provides us an opportunity towards unbiased screening of molecular changes that occur in Notch2 deregulated B cells and identification of downstream target genes and pathways. The outcome of the study could help us to focus on devising new alternative treatment strategies other than γ-secretase inhibitors, targeting pathways that are modulated in B-cell lymphoma.

## RESULTS

### Knockdown of Notch2 using shRNA

**Figure 1:**
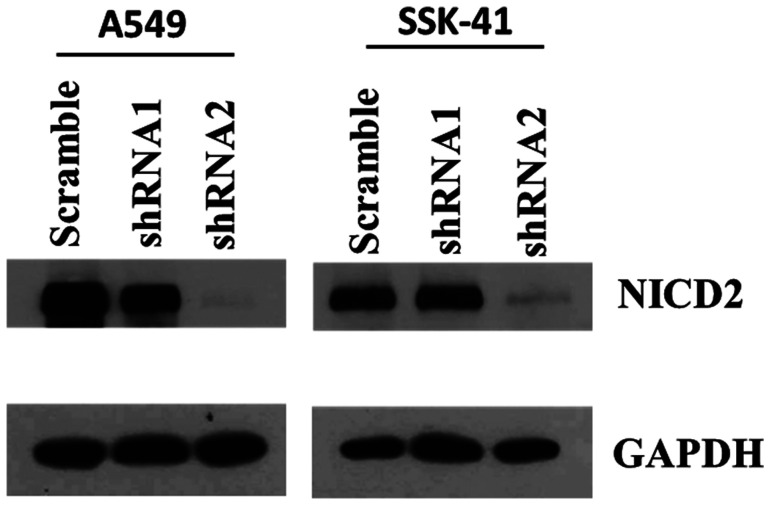
Efficiency of Notch2 knockdown in A549 and SSK-41 cells by lentiviral vectors bearing shRNA. Cells were infected with lentiviral vectors of Notch2-shRNA1, Notch2-shRNA2 and scrambled shRNA. The Notch2 protein expression levels were determined by Western blotting assays and GAPDH was used as a loading control.

To determine the efficacy of Notch2-shRNA in reducing the intracellular levels of Notch2, we treated A549 (lung cancer) and SSK-41 cells (B-cell lymphoma) with viral supernatants of two different shRNA constructs in a lentiviral vector targeting Notch2. A549, an adherent and easy to transduce cells were used in this experiment to validate the efficacy of the two different shRNA’s against Notch2 expression. Subsequent immunoblotting experiments revealed that both lentiviral vectors were able to reduce the levels of intracellular domain of Notch2 (NICD2), most noticeably Notch2-shRNA2 showed marked reduction in the Notch2 levels in A549 and SSK-41 cells (Figure 1). Therefore, Notch2-shRNA2 construct was used for further experiments.

### Determination of reproducibility of biological replicates

**Figure 2:**
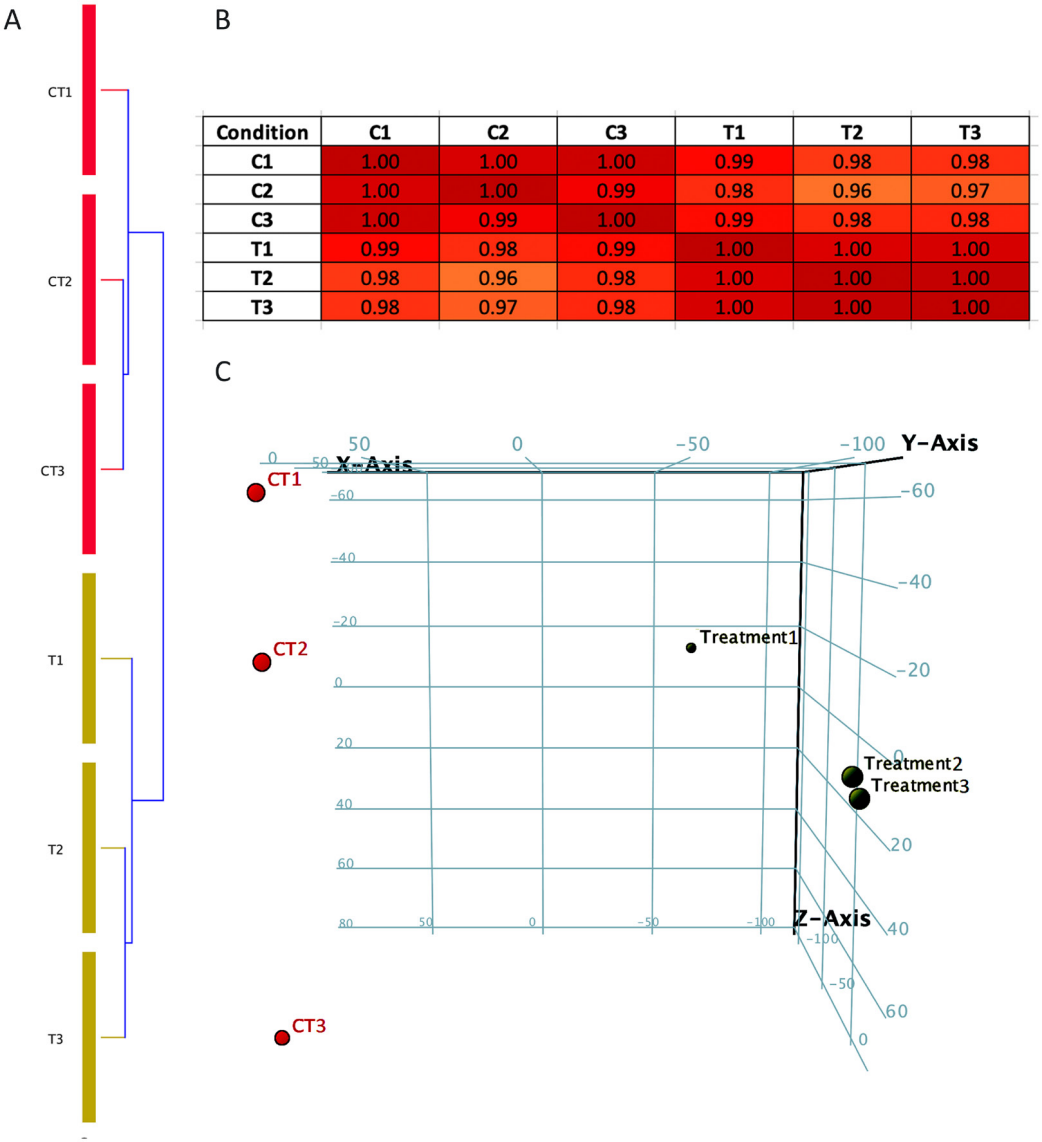
Condition tree, Correlation matrix and Principal component analysis (PCA plot). (**A**) Condition tree were plotted against Notch2-shRNA treated (T1, T2 & T3) and control samples (C1, C2 & C3). (**B**) Correlation matrix between treated (T1, T2 & T3) and control samples (C1, C2 & C3) indicating positive correlation between the samples. (**C**) Principal component analysis performed between Notch2-shRNA and control samples. The PCA plot is showed in a three dimension, where red colour sphere indicates control group and black colour sphere indicates Notch2-shRNA treated group.

The data obtained from RNA-Seq analysis were subjected to quality control (QC) parametric to ensure the quality and reproducibility of the assay. It is important to perform these tests in order to enhance the confidence of the results obtained using these data. Condition tree, correlation matrix and principal component analysis (PCA) were plotted using Cluster 3.0 tool [26, 27]. A condition tree, correlation matrix and principal component analysis (PCA) allows us to visualize similarities and differences within and between the Notch2-shRNA treated (T1, T2 & T3) and control samples (C1, C2 & C3). The results of correlation matrix and principal component analysis (PCA) of all samples (Figure 2A–2C) showed that samples were highly correlated. The PCA analysis found that treatment 1 sample (T1) to be outlier from T2 and T3 (Figure 2C) but with very little biological variation, which was plotted in correlation matrix table (Figure 2B). Overall, the results of all the three tests showed significant reproducibility among Notch2 knockdown and control groups.

### Identification of differentially expressed genes (DEGs)

**Figure 3:**
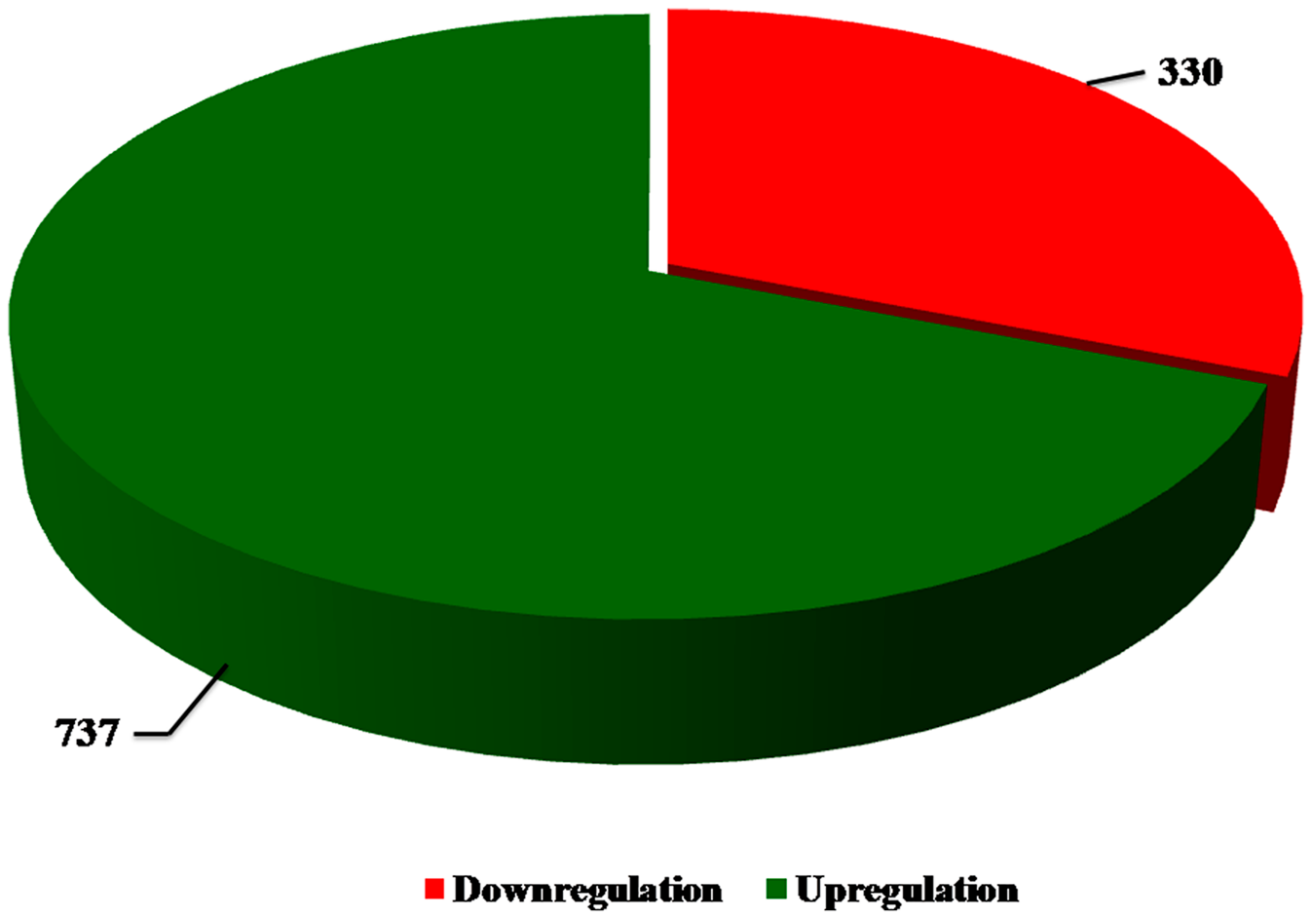
Pie chart. The number of upregulated and downregulated DEGs were identified between SSK-41 cells treated with scramble-shRNA or Notch2-shRNA. The green colour indicates the total number of upregulated DEGs and the red colour indicates the total number of downregulated DEGs.

Subjecting the transcriptomic data to bioinformatics analysis revealed several genes that are significantly altered in Notch2 depleted cells when compared to controls. Among them, only genes that showed highly significant changes were taken for further consideration. A pie chart shows the number of DEGs in Notch2-shRNA treated samples when compared with control samples (Figure 3). The results showed that 737 genes were upregulated and 330 genes were downregulated upon knockdown of Notch2.

**Figure 4:**
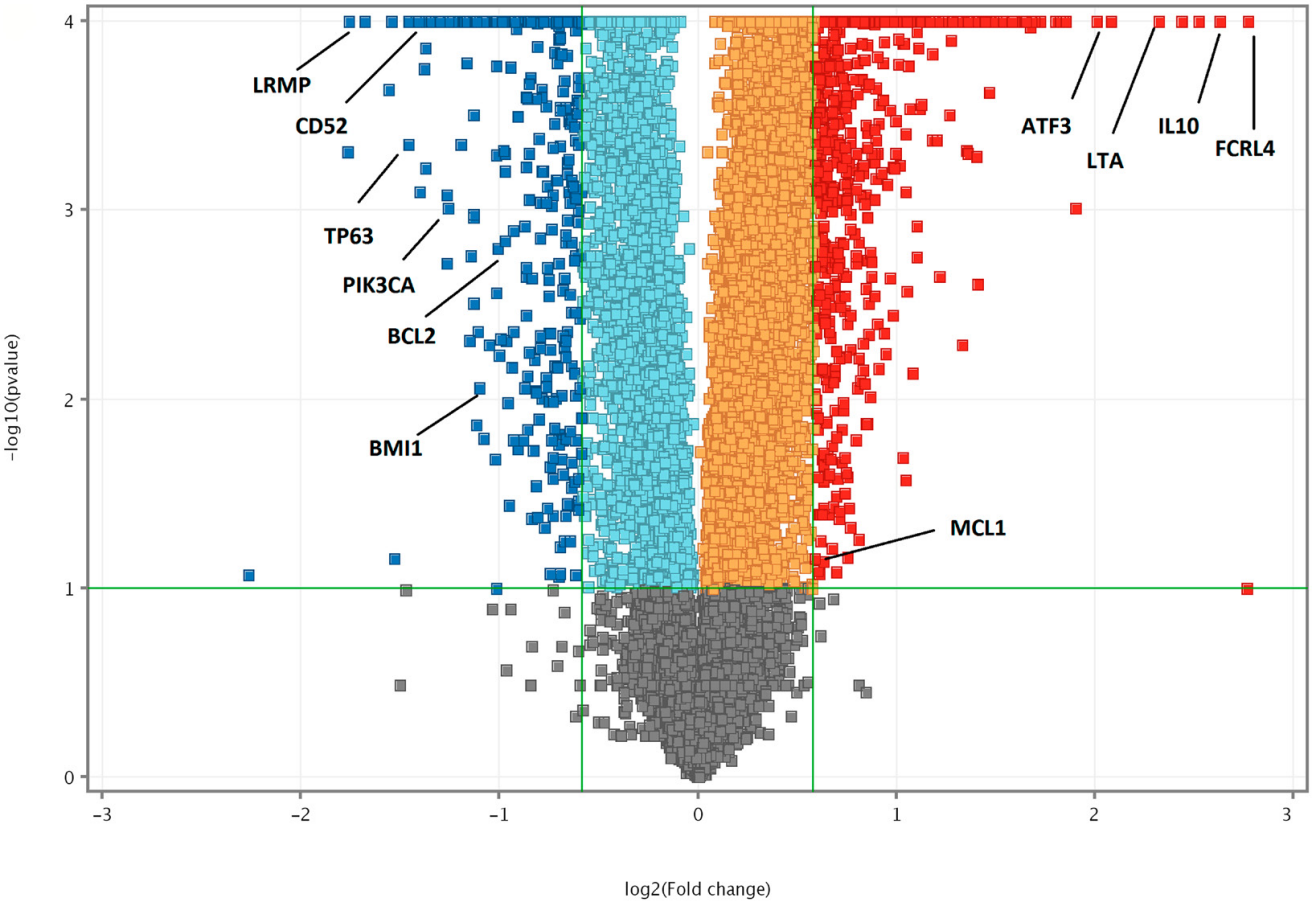
Volcano plot of DEGs (FDR < 0.05) between Notch2-shRNA and control. The horizontal axis is the log2 fold change between Notch2-shRNA and Control. The negative log10 of the *P*-value of Fisher’s exact test is plotted on the vertical axis. Each gene is represented by one point on the graph. Volcano plot highlighting top significant DEGs from Notch2-shRNA treated samples. Significantly upregulated genes are in blue colour; significantly downregulated genes are in red colour.

Further, we used a false discovery rate (FDR) < 0.05 and plotted them against the log2 fold change to determine the significant differences in gene expression [28]. We identified 727 genes, that were significantly upregulated and 320 genes that are significantly downregulated, which were represented in a volcano plot (Figure 4).

**Figure 5:**
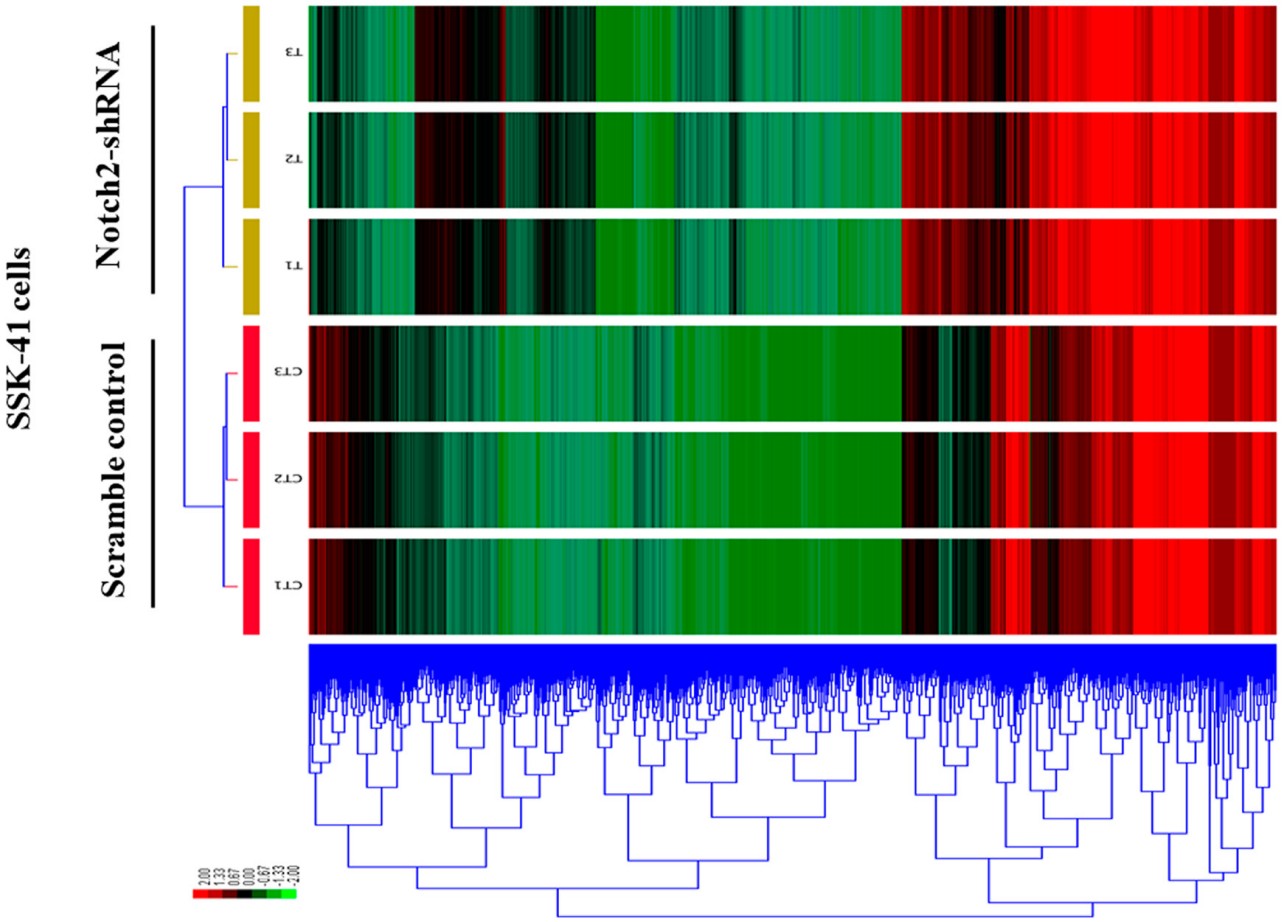
A clustered heat map showing the expression patterns of DEGs in control and Notch2-shRNA treated SSK-41 cells. The red blocks represent the overexpressed genes, and the green blocks represent genes with the lowest expression levels.

A clustered heat map shows the expression profiling of the DEGs in Notch2-shRNA treated samples when compared with control samples (Figure 5). Red blocks represent the overexpressed genes and the green blocks represents lowly expressing genes. The heat map clustering showed a distinguished gene expression pattern in Notch2-shRNA treated samples when compared to controls.

These significant analyses found that, 194 DEGs were observed in Notch2-shRNA treated samples when compared with control samples, keeping the threshold of fold change as > 2 [28]. Among the 194 DEGs, 136 DEGs were significantly upregulated and 58 DEGs were significantly downregulated (Table 1).

**Table 1:** Detailed information of the differentially expressed genes (DEGs) from the significant analyses result based on threshold of fold change > 2

DEGs	Gene names
**Upregulated**	*TIPARP, MYADML2, PIK3R3, RP11-638I2.8, TANGO6, SGK1, EHBP1, DNAJB1, RGS1, RPS6P7, B3GALNT1, TBC1D17, CBS, WIPI1, AC159540.1, ASS1, PALM2-AKAP2, RP11-429G19.3, CTH, MPEG1, RPP40, RP5-1120P11.4, RP11-507K12.1, SLC29A2, NPPA-AS1, PHTF1, WARS, TCAF2, MAD1L1, TREX2, RP11-326C3.2, NQO1, BRE, ZNRF1, RP11-20D14.6, DYX1C1-CCPG1, IFIT3, RP11-734K23.9, RP4-647J21.1, AP000648.5, CYP26A1, MTATP8P2, DHRS2, ZNF248, SMIM10, CHMP4C, CAPG, SP140, RNF213, GPR157, RP11-147G16.1, HSPA8, RP11-1348G14.8, PPP1R15A, MAP1A, MSMO1, B4GALNT1, GADD45B, TBC1D27, CHORDC1, DUSP1, AL135791.1, ANKRD34A, ENOX2, RP11-284N8.3, RASGRP1, DBET, IL4I1, ATF3, TRIM22, IDH1, DUSP5, IFI35, MT1F, TRIB3, C8G, PGAM1P7, DHCR24, ACACA, ABCA7, SNAI2, RPTOR, MCTS2P, DGKG, CTD-2537I9.12, EGR1, RP11-67L3.5, CHAC1, RP11-108L7.14, IL2RB, WFDC21P, HSPA1B, STX3, HSPA1A, RP11-567J20.3, KSR1, RXRA, DOK1, RP11-799B12.1, IFI6, RP11-122G18.7, ROCK1P1, RP11-325K4.2, LTA, LDLR, MT1G, SLC6A9, SLA, KLF6, TMOD1, ZBTB32, INHBE, APOL2, CTD-2342N23.3, AHR, RP11-625L16.3, C5AR1, RP11-271C24.2, EGR2, CTD-2060L22.1, AC009950.1, BATF3, CCL4L2, FOS, APOL1, AC002069.5, LINC00881, CD69, CCL3L3, TEX19, LOXL3, NCF2, IL10, FCRL4, RPS21P4, SOCS3*
**Downregulated**	*RP11-373L24.1, RP11-642A1.2, SERTAD4-AS1, HMCES, ASTL, KMT2E-AS1, B3GAT2, RP11-385F7.1, NUGGC, RP11-44F21.5, AC002310.11, RP5-855D21.3, LINC00115, CDKN1C, RP11-713N11.4, RP4-548D19.3, SNX29P1, LRMP, WDR7-OT1, CTB-31O20.2, SMIM18, RP11-66N11.8, RP11-837J7.4, KB-1460A1.5, H1FX, RP11-798M19.3, RP11-706P11.2, RP11-448G15.3, RP11-486G15.2, AC023590.1, RP11-477I4.4, AC006077.3, HIST1H4J, HIST2H2AA3, RP3-395M20.12, PYY2, HIST2H2AA4, SCG5, LINC01481, TTC28-AS1_1, RP11-381P6.1, HIST1H2AE. LA16c-329F2.2, GCSAM, SNORA73B, SLC12A4, TRERNA1, CD52, CTB-119C2.1, SNORD3A, HIST1H1C, RN7SL644P, TSPOAP1, MROH7-TTC4, HIST1H3H, HIST2H2BC, KB-1107E3.1, RP11-501C14.5*

A total of 194 DEGs were identified, including 136 upregulated genes and 58 downregulated genes.

### Distribution of DETs based on transcript type

**Figure 6:**
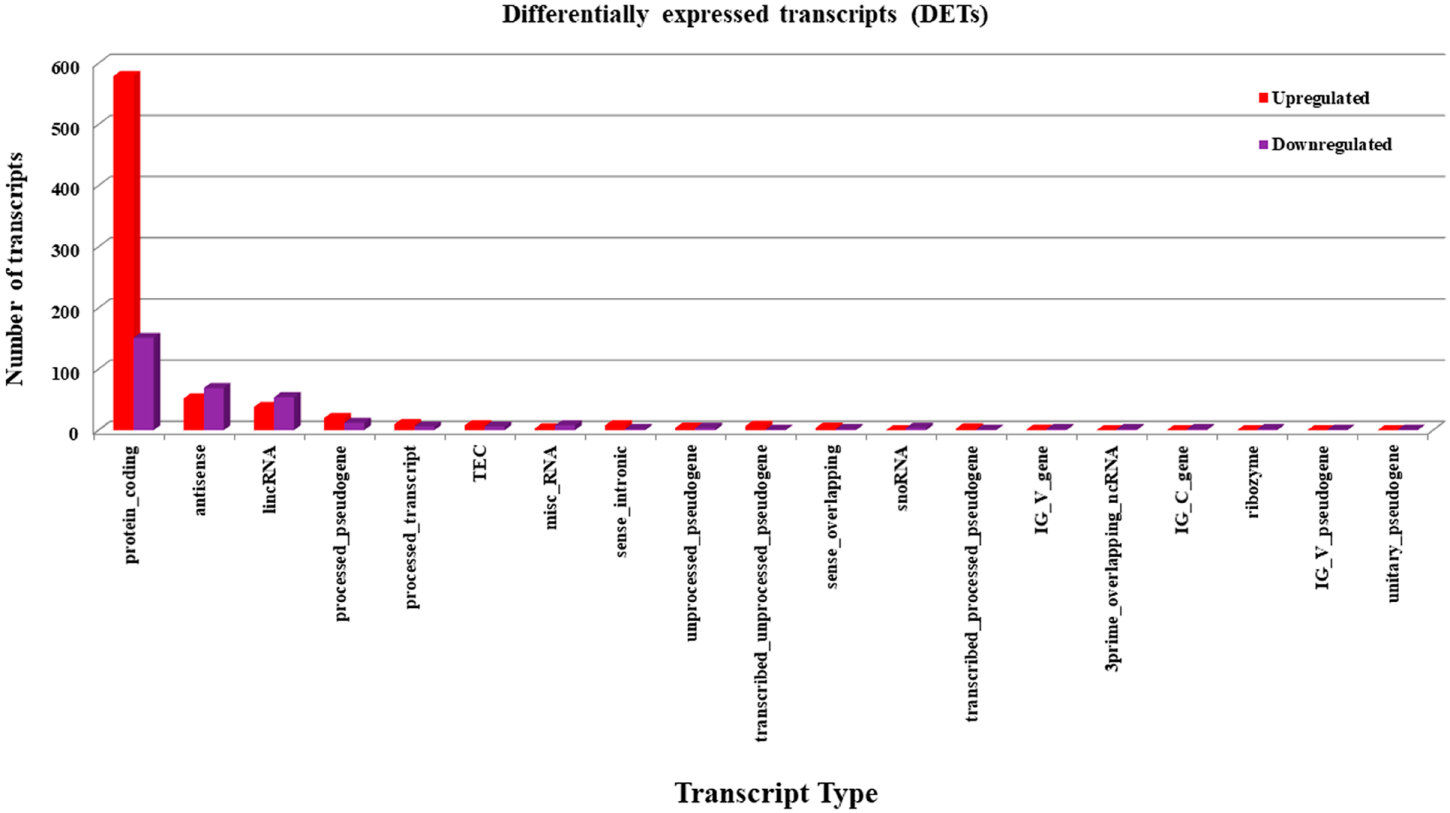
Identification of differentially expressed transcripts (DETs). Statistically significant, differentially expressed transcripts upon knockdown of Notch2 were identified with Cuff Diff pipeline with a fold change cutoff of ≥ 1.5, and a student *t*-test *P*-value of ≤ 0.05. Red colour indicates the upregulated DETs and violet colour indicates downregulated DETs.

Mammalian cells express several forms of transcripts ranging from protein coding to long non-coding RNAs. The expression levels of these different types of RNA transcripts from Notch2-shRNA treated samples were compared with control samples. The differentially expressed transcripts from treated samples were identified using Cuff Diff pipeline with a fold change cutoff of ≥ 1.5 and a student *t*-test *P*-value of ≤ 0.05 upon treatment [29, 30]. From this analysis, we have identified 1,067 statistically significant transcripts. Among these 737 transcripts were found to be upregulated and 330 transcripts were found to be downregulated (Figure 6). The results show that, knockdown of Notch2 resulted in a net increase in the expression of transcripts, indicating a possible induction of transcription in these cells.

### GO enrichment analysis of DEGs

To gain further insight into the potential functions and their role in the metabolic pathways, the identified DEGs were subjected to gene enrichment analysis using DAVID tool [31]. GO (Gene Ontology) term enrichment analysis resulted in unique 208 GO categories and pathways. Furthermore, the GO categories were classified into different functional categories according to the GO term enrichment analysis for biological process (BP), molecular function (MF) and cellular component (CC). Only significant GO categories with *P*-values ≤ 0.05 were chosen for further analysis. Among the 208 GO categories, 31 pathways were significantly enriched in biological processes (BP), 3 pathways were significantly enriched in cellular components (CC) and 18 pathways were significantly enriched in molecular functions (MF).

**Figure 7:**
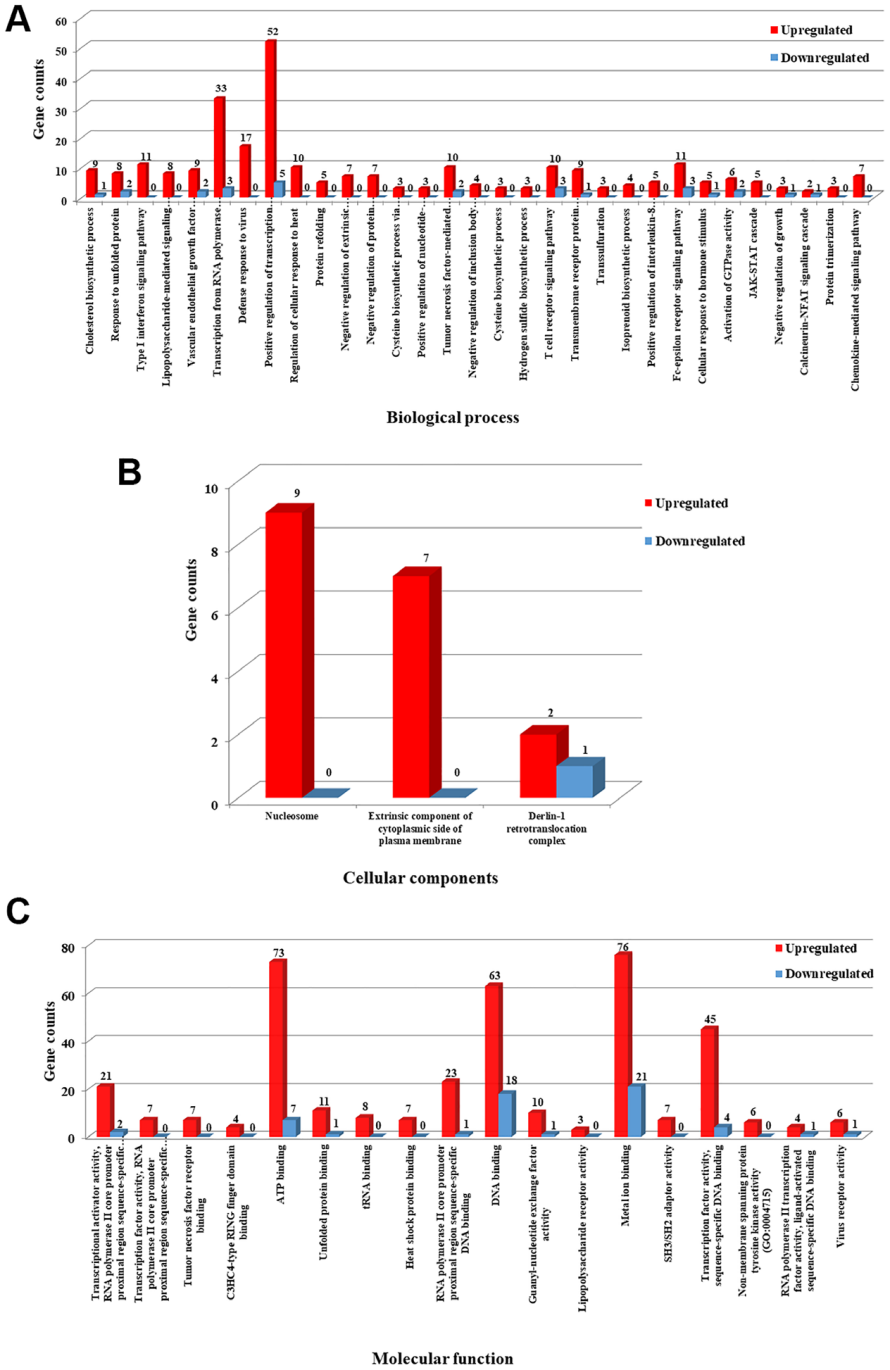
GO terms enrichment analysis of DEGs. Statistically significant GO enrichment analysis of DEGs was identified with *P*-values ≤ 0.05 in different functional groups. (**A**) GO analysis results of upregulated and downregulated DEGs were significantly enriched in biological process. (**B**) GO analysis results of upregulated and downregulated DEGs were significantly enriched in cellular component. (**C**) GO analysis results of upregulated and downregulated DEGs were significantly enriched in molecular function. Number in the bar represents count of DEGs enriched in corresponding GO classification.

For biological processes (Figure 7A), the upregulated DEGs were mainly enriched in positive regulation of transcription from RNA polymerase II promoter, defense response to virus, type I interferon signaling pathway, Fc-epsilon receptor signaling pathway, T cell receptor signaling pathway and tumor necrosis factor-mediated signaling pathway. The downregulated DEGs were also enriched in positive regulation of transcription from RNA polymerase II promoter, transcription from RNA polymerase II promoter, T cell receptor signaling pathway, Fc-epsilon receptor signaling pathway, vascular endothelial growth factor receptor signaling pathway, activation of GTPase activity and response to unfolded protein. Regarding cellular components (Figure 7B), the upregulated DEGs were enriched in nucleosome and extrinsic component of cytoplasmic side of plasma membrane and the downregulated DEGs were primarily enriched in the Derlin-1 retrotranslocation complex. For molecular functions (Figure 7C), the upregulated DEGs were significantly enriched in ATP binding, DNA binding, metal ion binding and transcription factor activity, sequence-specific DNA binding and the downregulated DEGs were enriched in metal ion binding, DNA and ATP binding activities. All the detailed GO term enrichment analysis results are shown in Figure 7A–7C. Significant results of the GO enrichment analysis of DEGs were listed in Supplementary Tables 1 and 2.

### Significantly enriched pathway terms in DEGs

**Figure 8:**
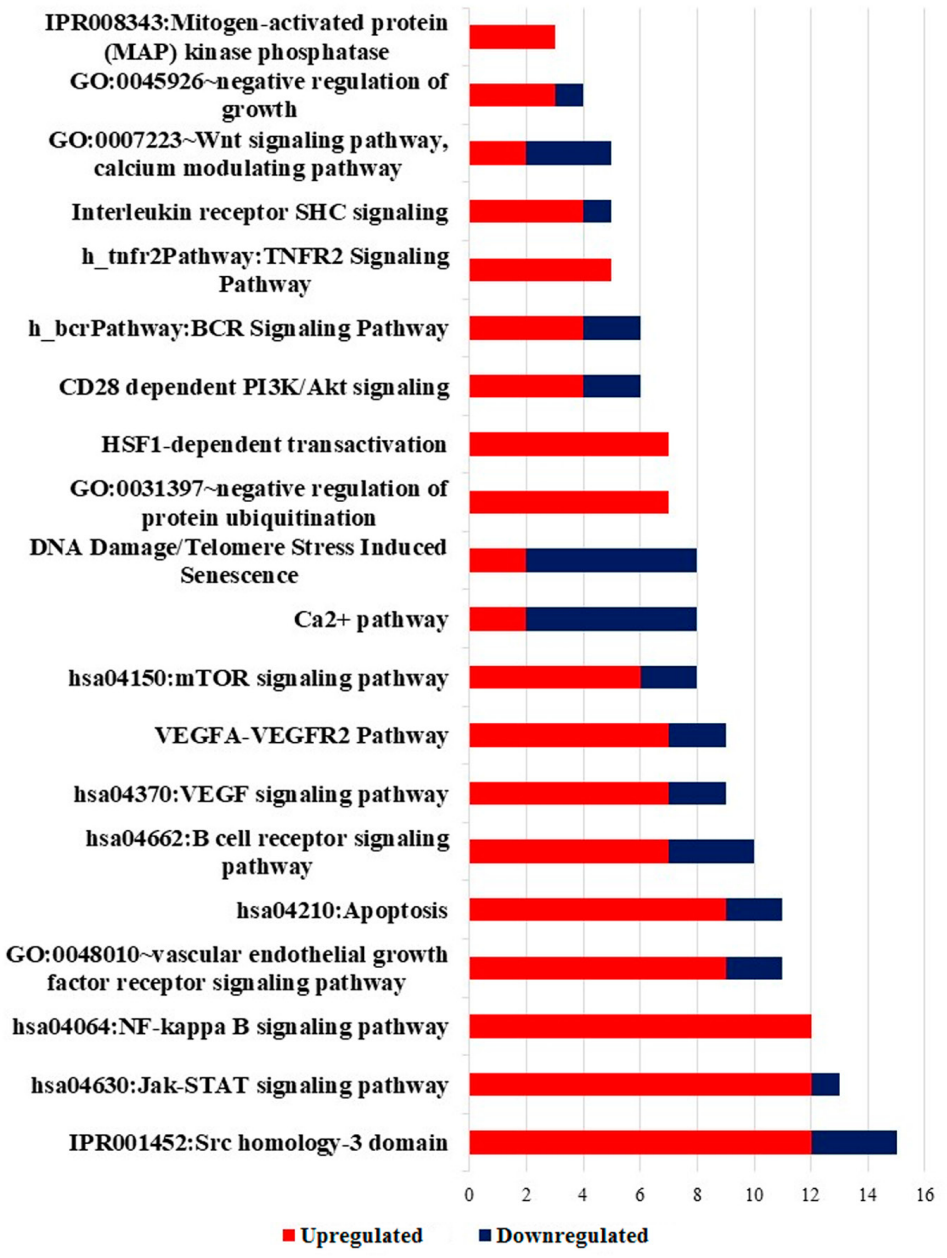
Significantly enriched pathway terms in DEGs. Significant enriched pathway terms in DEGs identified in Notch2-shRNA treated samples. Red denotes upregulation and blue denotes downregulation of DEGs.

Further, in the list of DEGs obtained from Notch2-shRNA treated samples, the most significantly enriched pathways of the DEGs were identified using DAVID tool [31]. Only GO and pathways with FDR score of *≤* 0.05 were considered for downstream analysis. Phenotype vs Genotype analysis was done to identify statistically significant and enriched GO and pathways, which resulted in identification of 20 non-redundant GO and other pathways (Figure 8). The results showed that DEGs were highly clustered in several signaling pathways such as NF-kappaB signaling pathway, Jak-STAT signaling pathway, apoptotic pathway, B-cell receptor signaling pathway, mTOR signaling pathway, Ca^2+^ pathway, Wnt signaling and PI3K/AKT pathways. List of significantly modified genes from selected GO terms with fold change ≥ 1 and ≤ -1 were listed in Supplementary Table 3.

### Gene regulatory network analysis

**Figure 9:**
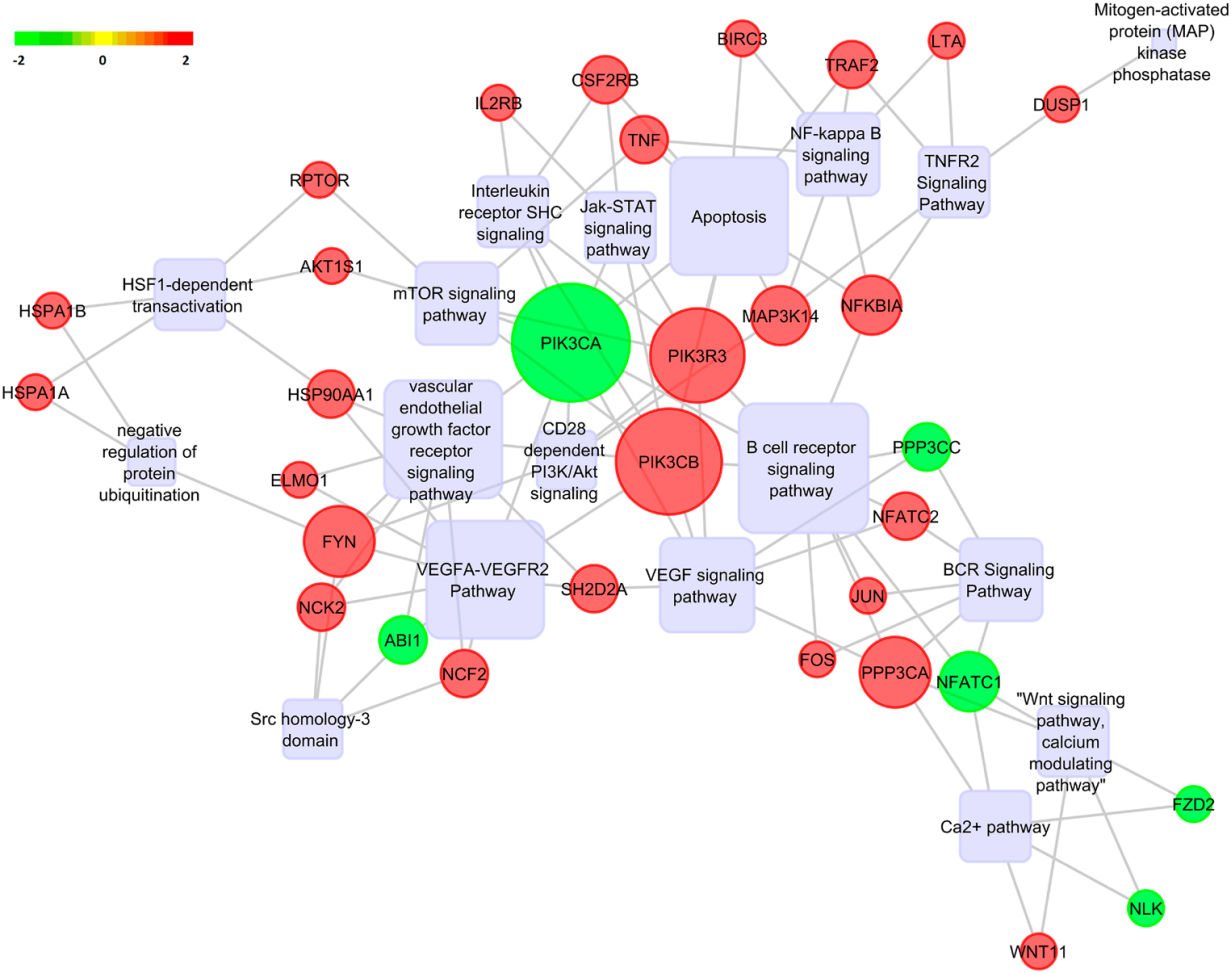
Pathway analysis. Gene regulatory network analysis for DEGs upon Notch2 knockdown were predicted by Pathreg algorithm and visualized in Cytoscape v2.8.2. Predicted pathways are depicted as rounded rectangle, where shades in red correspond to upregulated genes and shades in green correspond to downregulated genes.

The top 20 significantly enriched terms along with the differentially expressed genes within them was further subjected to gene regulatory network analysis using Pathreg algorithm (Theomics International Pvt Ltd, Bangalore). The resultant nodes and edges were visualized using Cytoscape v2.8.2 [32] to identify key nodes and edges that could be representative of the gene regulatory changes upon Notch2 knockdown. The results are shown in Figure 9. The results show that knockdown of Notch2 had profound effects on multiple important cellular pathways. The rounded rectangle shows the significant pathways in the gene regulatory network. The red colour sphere indicates the upregulated genes and green colour sphere indicates the downregulated genes. The size of the gene within the sphere is determined by the number of interconnecting nodes between different pathways and the genes of the regulatory network. Increased size of the gene corresponds to most number of interconnected pathways and genes. From the gene regulatory network, PIK3CA was found to be downregulated and acting as a hub between many interconnected pathways. The members of PI3K/AKT and NF-kB pathways, that function downstream of Notch2 were found to be downregulated, indicating that Notch2 might mediate its oncogenic effects by positively modulating these pathways.

### Validation of DEGs by qRT-PCR

**Figure 10:**
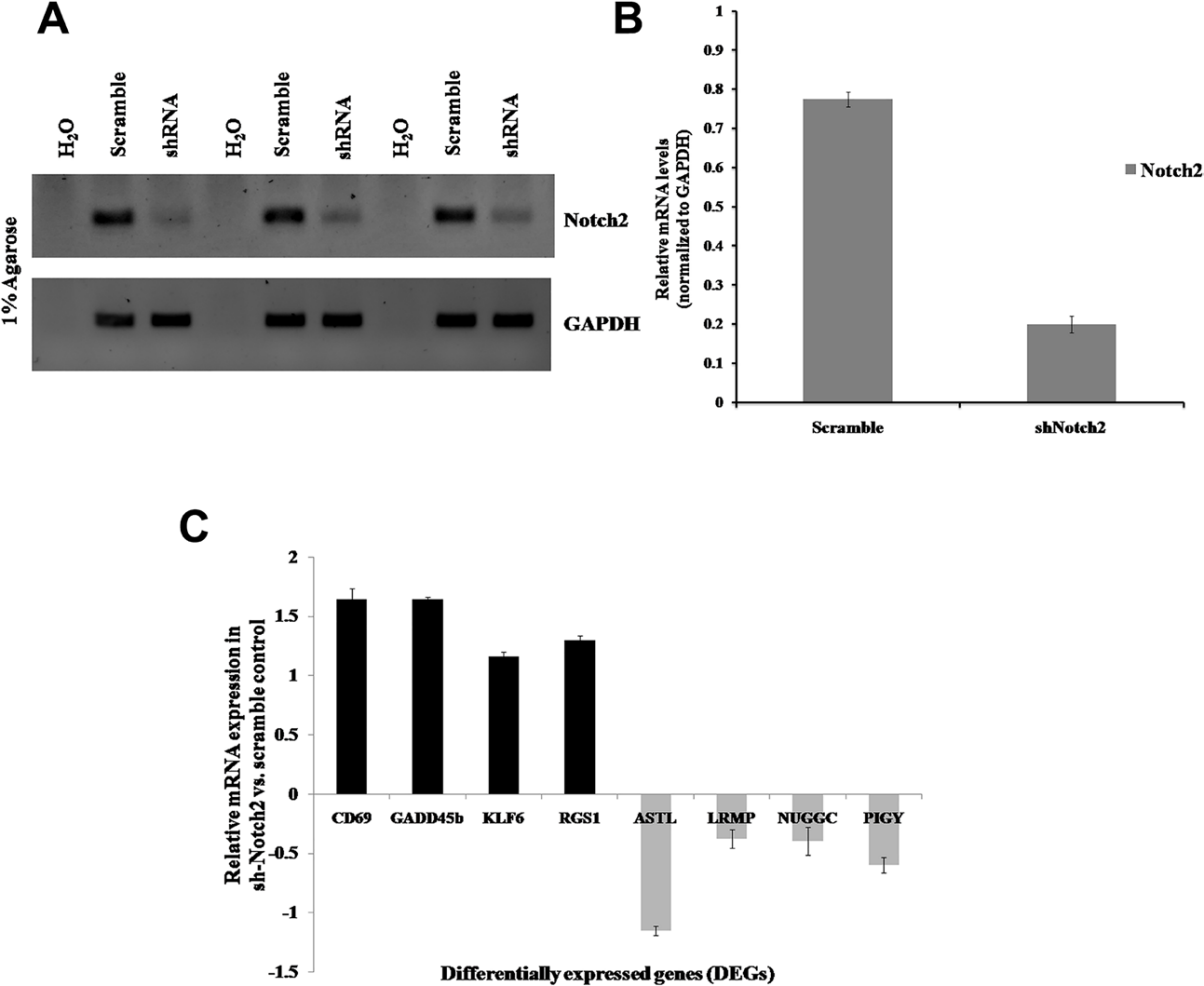
Validation of DEGs in B-cell lymphoma (JM1). JM1 cells were transduced with Notch2-shRNA2 or scrambled shRNA viral particles. (**A**) The Notch2 mRNA expression levels were determined by qRT-PCR, and H_2_O was used as a negative control. (**B**) Notch2 mRNA expression levels were quantified using ImageJ software and normalized with GAPDH. (**C**) qRT-PCR assay was performed to validate upregulated and downregulated DEGs in JM1 cells and normalized with GAPDH (Internal control). mRNA expression levels were quantified using ImageJ software and the bars were represented as mean ± SD. The assays were performed in triplicate experiments and values were plotted against scramble control.

To further confirm and validate the DEGs from the RNA-Seq data, total RNA were isolated from control and Notch2 knockdown JM1 cells, and qRT-PCR analysis was performed (Figure 10A and 10B). The upregulated and downregulated DEGs were selected based on fold change (log2) ≥ 2 and (log2) ≤–2. The selected upregulated genes were *CD69*, *GADD45b*, *KLF6*, *RGS1* and the downregulated genes were *ASTL*, *LRMP*, *NUGGC* and *PIGY,* which were validated by qRT-PCR analysis. Overall, the qRT-PCR results showed good correspondence with the RNA-Seq results, indicating that our results corroborate the RNA-Seq data with high reliability and accuracy (Figure 10C).

## DISCUSSION

RNA Sequencing (RNA-Seq) is a high-throughput sequencing technology utilized to obtain large quantities of transcriptome data from different organisms, which provides an efficient way to study gene expression on a genome-wide scale [33]. RNA-Seq is a more sensitive technology than expression profiling analysis using arrays, due to their low sensitivity and cross-hybridization of probes and targets [34]. In the present study, whole transcriptome analysis was performed in B-cells, where Notch2 expression is knocked down using Notch2-shRNA and compared with control scramble-shRNA treated cells. The RNA-Seq and bioinformatics technology revealed notable information regarding gene expression at the transcriptome level and identified multiple significant molecular pathways in response to knockdown of Notch2.

The significant reproducibility, similarity and distance between the treated and untreated group were identified using condition tree, correlation matrix and principal component analysis tests. Many pivotal genes and pathways associated with B-cell lymphoma were identified in the present study. In the first step, a total of 15,083 differentially expressed genes were identified between Notch2-shRNA treated and control samples. The identified DEGs were represented in a pie chart, which shows 737 genes to be upregulated and 330 genes downregulated upon Notch2 knockdown. The volcano plot highlighted the significant DEGs that were modulated after knockdown of Notch2 in B-cell lymphoma (727 upregulated and 320 downregulated). The clustered heatmap identified the expression profiling of the DEGs upon Notch2-shRNA knockdown. Many differentially expressed transcripts (DETs) were also identified that showed a possible induction in transcriptional regulation. In the second step, from the identified DEGs, a gene enrichment analysis was performed using DAVID tool, which resulted in identification of unique 208 GO categories and pathways. These GO terms were classified into three functional categories namely, biological process (BP), cellular component (CC) and molecular function (MF). The most significantly upregulated DEGs were involved in the different GO terms such as, regulation of transcription, apoptosis, ubiquitination, cell cycle, DNA replication, DNA repair and immune response mechanisms. Hence, these significantly enriched terms could help us to further understand which of the DEGs and DETs played the causative role in B-cell lymphomagenesis. Through further analysis, the upregulated and downregulated DEGs along with GO terms were further clustered based on functions and signaling pathways with significant enrichment analysis to develop a gene regulatory network. The validation of upregulated and downregulated DEGs at the mRNA levels by qRT-PCR analysis also provide additional support for their potential role in B-cell lymphoma.

The results of our gene network analysis suggest that, knockdown of Notch2 modulates multiple important cellular pathways, including immune-related pathways, apoptotic related pathway, PI3K/AKT, BCR, mTOR, VEGF, Wnt and Ca^2+^ signaling pathways. Among the above mentioned pathways, the NF-kB signaling is a major prosurvival pathway, which also has the ability to cross-talk with other survival pathways including PI3K/AKT in various cancers [35]. Aberrant activation of PI3K/AKT signaling pathway has been implicated in various hematologic malignancies including B-cell lymphomas [36]. Emerging reports have revealed that constitutive activation of NF-kB signaling pathway promotes continuous lymphocyte proliferation and survival, and has been considered as an important pathogenic factor in many subtypes of human B-cell lymphoma [37]. Molecular cross-talk between NF-kB and PI3K/AKT signaling pathway, and therapeutic effects of suppressing NF-kB activity by inhibiting PI3K signaling has been previously reported in human Burkitt’s lymphoma and diffuse large B-cell lymphoma [38, 39]. The present study also revealed that when Notch2 is depleted, several genes that tightly control cell cycle are found to be modulated along with downregulation of PI3K/AKT pathway, indicating these genes as probable downstream targets. Since activation of PI3K/AKT pathway is known to promote cell proliferation, cell survival, growth and angiogenesis in cancers [40], it is important to know if Notch2 propels cancer progression through activation of this pathway. However, the exact mechanism by which Notch2 regulates NF-kB activity via activating PI3K/AKT and inhibits apoptosis in B-cell lymphoma need to be determined. Nevertheless, establishing the role of PI3K/AKT pathway in Notch2 activated cancers could be very important to consider it as an alternative treatment target in mitigating the effects of Notch2 transactivity in these cancers. The detailed functional importance and regulatory role played by these differentially expressed genes and pathways in promoting B-cell lymphoma will be carefully elucidated in subsequent studies.

## MATERIALS AND METHODS

### Cell line maintenance

Human embryonic kidney cells (HEK293T) and human lung cancer cells (A549) were purchased from NCCS, Pune and cultured in Dulbecco’s Modified Eagle’s Medium (Himedia). Human splenic marginal zone lymphoma SSK-41 cell line was established and cultured in RPMI 1640 medium (Himedia) and B-cell lymphoma cells (JM1) was purchased from NCCS, Pune and cultured in Iscove’s Modified Dulbecco’s Medium (Himedia) supplemented with 10% fetal bovine serum (Gibco, Thermo fisher Scientific, Waltham, MA, USA) and 1% penicillin/streptomycin solution in a humidified incubator at 37°C and a 5% of CO_2_ atmosphere [41].

### Lentiviral vector production and transduction

Lentiviral vector pLKO.1-puro (Sigma-Aldrich) expressing a short hairpin RNA (shRNA) targeting human Notch2 gene and lentiviral scrambled shRNA control plasmids were constructed. The lentiviral vectors were co-transfected with pMD2.G and psPAX2 packaging plasmids in HEK293T cells with Polyjet™ transfection reagent (SignaGen Laboratories, Gaithersburg, MD, USA) according to the manufacturer’s instructions. After 24 hrs of post transfection, the supernatants were collected and the cell debris was removed by centrifuging at 1600 rpm for 5 mins. The viral particles were harvested by centrifuging at 14,000 rpm for 2 hrs at 4°C. The viral particles concentrated as sediments on the walls of the tubes, were then collected and used for transduction. For transduction, the viral particles were resuspended with 5 ml of RPMI-1640 media at a concentration of 5X. The cells to be infected were seeded in 6-well plates and were infected with 200 μl of viral particles and 400 μl viral supernatant along with 4 μg/ml of polybrene. After 18 hrs infection, the cells were resuspended and maintained in fresh media for another 4 days. Notch2-shRNA stable cell lines were established by transducing SSK-41, A549 and JM1 cells with purified virus and stable pools of cells were selected in the presence of 350 ng/mL of puromycin (G-Bioscience, USA) [42, 43]. The Notch2-shRNA sequences were listed in Supplementary Table 5.

### Western blotting

Cells were lysed in 1X lysis buffer containing protease and phosphatase inhibitors. Equal amounts of protein lysates were separated by SDS-PAGE and transferred onto nitrocellulose membrane (Amersham, GE Healthcare Life Sciences, Inc.). The membranes were then incubated with specific primary antibodies Notch2 (Cell Signaling Technology, USA) and GAPDH (Bio-Rad, USA) at 4°C overnight. This was followed by incubation with HRP-conjugated secondary antibody (Bio-Rad, USA). Immunoblotting signals were visualized using the ImageQuant™ LAS 500 detection system (GE Healthcare Life Sciences, Inc., Chicago, IL, USA) [44].

### RNA isolation and sequencing

Total RNA was isolated from control and Notch2 knockdown SSK-41 cell line using Trizol reagent (Invitrogen), according to the instructions of manufacturer. The quality and quantity of the total RNA were evaluated using a NanoDrop2000 spectrophotometer. The RNA samples used for transcriptome analysis were further subjected to quality check by using Agilent 2100 bio-analyzer system. The RNA samples with RNA integrity number (RIN) > 6 were used for further analysis. For each sample, 10 μg total RNA was used for RNA-Seq library preparation. The NextSeq 500 system (Illumina, Inc., San Diego, CA, USA) was used to collect data from triplicate of scramble control and Notch-2 knockdown samples [45].

### Bioinformatics analysis

Raw data were obtained in FASTq format for the triplicate samples with a read length of 100 bp, which on an average provided 7.5 million HQ reads per sample upon quality control analysis using NGSQC ToolKit [46]. Further, the HQ passed reads were subjected to splice var alignment against Human Genome Build GRCh38 using TopHat aligner [47, 48]. Analysis of replicate reproducibility was done by plotting Condition tree, PCA (Principal Component Analysis) and Correlation Matrix using Cluster 3.0 tool [26, 27].

### Biological analysis of DEGs (differentially expressed genes)

Differentially expressed genes (DEGs) were detected using package edge R [49]. Differentially expressed genes (DEGs) between Notch2-shRNA and control samples were identified using the false discovery rate (FDR) < 0.05 and plotted against the log2 fold change and threshold of fold change > 2 [28].

### Pathway regulatory network modelling of DEGs

Statistically significant differentially expressed transcripts were subjected to GO and Pathway enrichment using DAVID tool [31]. Only those GO and pathways with a FDR score of ≤ 0.05 was considered for further downstream analysis. The GO and pathway terms along with the differentially expressed genes within them was subjected to gene regulatory network analysis using Pathreg algorithm from Theomics International Pvt Ltd, Bangalore, India. The resultant nodes and edges were visualized using Cytoscape v2.8.2 [32].

### Validation of DEGs by qRT-PCR analysis

Quantitative reverse transcription polymerase chain reaction (qRT-PCR) was performed to validate upregulated and downregulated DEGs that were selected based on the criteria: DEGs with a fold change (log2) ≥ 2 and (log2) ≤-2. The total RNA were isolated from scramble control and Notch2 knockdown JM1 cell lines using Trizol reagent (Invitrogen) according to the manufacturer instructions. Total RNA (2000 ng) was converted to cDNA using Superscript III (Invitrogen) according to the manufacturer instructions. GAPDH was used as internal control. PCR products were resolved by 1% agarose gel electrophoresis and visualized by ImageQuant™ LAS 500 (GE Healthcare Life Sciences, Inc., USA) [50]. The primer sequences were listed in Supplementary Table 4. All data are presented as the mean ± standard deviation (SD) performed in triplicate experiments [51].

## SUPPLEMENTARY MATERIALS






