# Supplementary material for: RNA sequencing analyses reveal differentially expressed genes and pathways as Notch2 targets in B-cell lymphoma
Source: Oncotarget. 2020 Dec 1;11(48):4527–40. doi: 10.18632/oncotarget.27805 (PMC7721612; doi:10.18632/oncotarget.27805)
Supplement: Supplementary file 1 [file oncotarget-11-4527-s001.pdf]

RNA sequencing analyses reveal differentially expressed genes and pathways as Notch2 targets in B-cell lymphoma

SUPPLEMENTARY MATERIALS

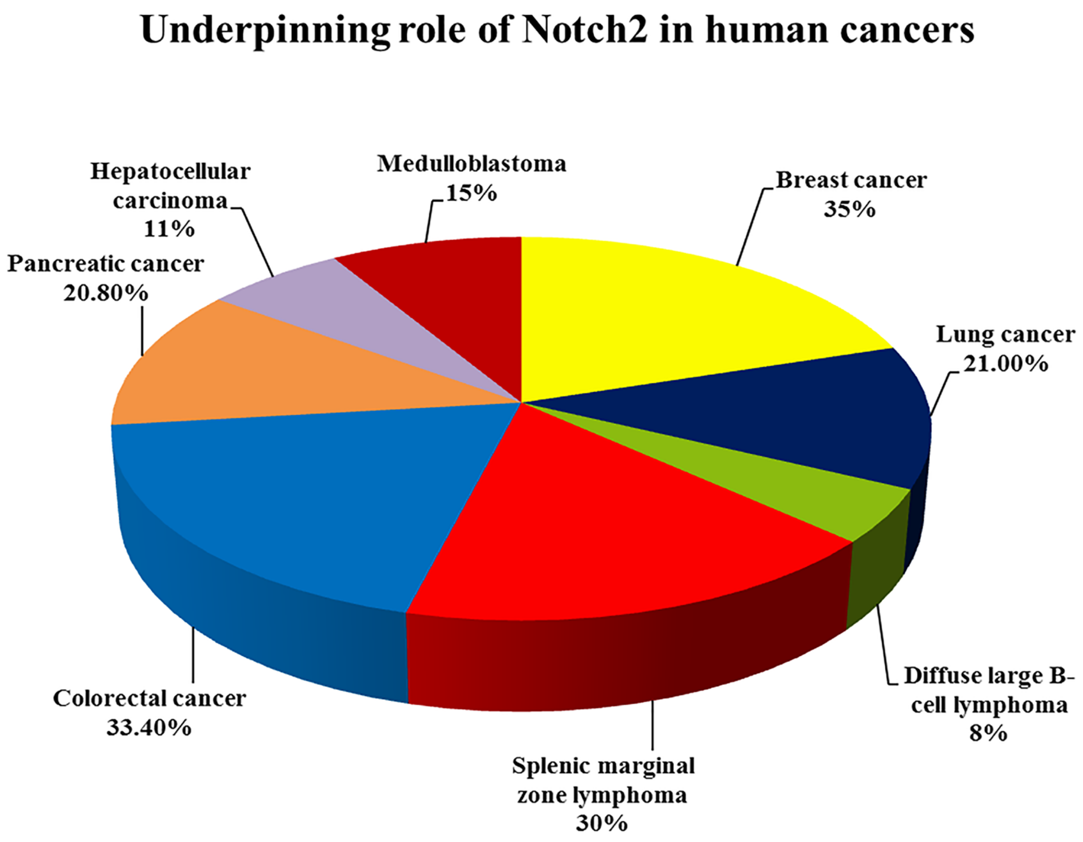

Supplementary Figure 1: The underpinning role of Notch2 deregulation in various human cancers.

Supplementary Table 1: GO enrichment analysis of significantly upregulated DEGs (*P*-values ≤ 0.05) were enriched in biological processes (BP), molecular functions (MF) and cellular components (CC). See Supplementary Table 1

Supplementary Table 2: GO enrichment analysis of significantly downregulated DEGs (*P*-values ≤ 0.05) enriched in biological processes (BP), molecular functions (MF) and cellular components (CC)

DEGs	Term	Description	Category	*P*-value
**Downregulated**	GO:0006695	Cholesterol biosynthetic process	BP	1×10^{-4}
	GO:0006986	Response to unfolded protein	BP	1×10^{-4}
	GO:0048010	Vascular endothelial growth factor receptor signaling pathway	BP	1×10^{-4}
	GO:0006366	Transcription from RNA polymerase II promoter	BP	1×10^{-4}
	GO:0045944	Positive regulation of transcription from RNA polymerase II promoter	BP	1×10^{-3}
	GO:0033209	Tumor necrosis factor-mediated signaling pathway	BP	5×10^{-3}
	GO:0007169	Transmembrane receptor protein tyrosine kinase signaling pathway	BP	1.0×10^{-2}
	GO:0038095	Fc-epsilon receptor signaling pathway	BP	1.6×10^{-2}
	GO:0032870	Cellular response to hormone stimulus	BP	2.5×10^{-2}
	GO:0090630	Activation of GTPase activity	BP	2.8×10^{-2}
	GO:0045926	Negative regulation of growth	BP	3.2×10^{-2}
	GO:0033173	Calcineurin-NFAT signaling cascade	BP	3.4×10^{-2}
	GO:0036513	Derlin-1 retrotranslocation complex	CC	5×10^{-2}
	GO:0001077	Transcriptional activator activity, RNA polymerase II core promoter proximal region sequence-specific binding	MF	1×10^{-4}
	GO:0005524	ATP binding	MF	1×10^{-3}
	GO:0051082	Unfolded protein binding	MF	3×10^{-3}
	GO:0000978	RNA polymerase II core promoter proximal region sequence-specific DNA binding	MF	8×10^{-3}
	GO:0003677	DNA binding	MF	1.2×10^{-2}
	GO:0005085	Guanyl-nucleotide exchange factor activity	MF	1.3×10^{-2}
	GO:0046872	Metal ion binding	MF	1.4×10^{-2}
	GO:0003700	Transcription factor activity, sequence-specific DNA binding	MF	2.4×10^{-2}
	GO:0004879	RNA polymerase II transcription factor activity, ligand-activated sequence-specific DNA binding	MF	4.4×10^{-2}
	GO:0001618	Virus receptor activity	MF	4.6×10^{-2}

Supplementary Table 3: List of significantly modified genes from selected GO terms with fold change ≥1 and ≤-1. See Supplementary Table 3

Supplementary Table 4: Primers used for qRT-PCR

S. No	Gene	Primer Sequence	Product Length
1.	*Notch2*	**F**-5′- ACAGTTGTGTCTGCTCACCAGGAT-3′<br>**R**-5′- GCGGAAACCATTCACACCGTTGAT-3′	119 bp
2.	*GAPDH*	**F**-5′- CATGACCACAGTCCATGCCAT-3′<br>**R**-5′- AAGGCCATGCCAGTGAGCTTC-3′	175 bp
3.	*ASTL*	**F**-5′-CCTTGCCAGGTGTGATCCTA-3′<br>**R**-5′-TTGCAGGAATGTCCTTGTCC-3′	126 bp
4.	*LRMP*	**F**-5′- GGCTGCATCAGGATGAATGA-3′<br>**R**-5′- TCTCCTGTCAAGATCATGGGT-3′	158 bp
5.	*NUGGC*	**F**-5′- GCCTTGATTGAAAAGCCGAC-3′<br>**R**-5′- TGGTCAGACAGAAGGTGGAT-3′	209 bp
6.	*PIGY*	**F**-5′- TCCAAAGGAGTGATGTTGGAAT-3′<br>**R**-5′- AACGACTACTGCAAACTGGT-3′	213 bp
7.	*CD69*	**F**-5′- TGGTTCAACGTTACAGGGTC-3′<br>**R**-5′- GAAGACTTCGGACCACAGAG-3′	200 bp
8.	*GADD45b*	**F**-5′- TCTGCTGTGACAACGACATC-3′<br>**R**-5′- TGTGAGGGTTCGTGACCA-3′	138 bp
9.	*KLF6*	**F**-5′- TTTAACGGCTGCAGGAAAGT-3′<br>**R**-5′- GCATTTAAAAGGCTTGGCCC-3′	177 bp
10.	*RGS1*	**F**-5′- TCCCTGGCTGAAGGGAATTA-3′<br>**R**-5′- TCTGCGCCTGGATAACTTTC-3′	158 bp

Supplementary Table 5: List of Notch2-shRNA sequence

S. No	Description	Target sequence
1.	Notch2-shRNA1	CCGGCCCACTAATAAGTGGTACTATCTCGAGATAGTACCACTTATTAGTGGGTTTTT
2.	Notch2-shRNA2	CCGGCCAGGATGAATGATGGTACTACTCGAGTAGTACCATCATTCATCCTGGTTTTT
