# Supplementary material for: RNA sequencing analyses reveal differentially expressed genes and pathways as Notch2 targets in B-cell lymphoma
Source: Oncotarget. 2020 Dec 1;11(48):4527–40. doi: 10.18632/oncotarget.27805 (PMC7721612; doi:10.18632/oncotarget.27805)
Supplement: Supplementary file 2 [file oncotarget-11-4527-s002.docx]

**Supplementary Table 1: GO enrichment analysis of significantly upregulated DEGs (*P-*values** *≤* **0.05) were enriched in biological processes (BP), molecular functions (MF) and cellular components (CC)**

| **DEGs** | **Term** | **Description** | **Category** | **P‑value** |
| --- | --- | --- | --- | --- |
| **Upregulated**  **Upregulated**  **Upregulated**  **Upregulated** | GO:0006695 | Cholesterol biosynthetic process | BP | 1 × 10^-4^ |
|  | GO:0006986 | Response to unfolded protein | BP | 1 × 10^-4^ |
|  | GO:0060337 | Type I interferon signaling pathway | BP | 1 × 10^-4^ |
|  | GO:0031663 | Lipopolysaccharide-mediated signaling pathway | BP | 1 × 10^-4^ |
|  | GO:0048010 | Vascular endothelial growth factor receptor signaling pathway | BP | 1 × 10^-4^ |
|  | GO:0006366 | Transcription from RNA polymerase II promoter | BP | 1 × 10^-4^ |
|  | GO:0051607 | Defense response to virus | BP | 1 × 10^-4^ |
|  | GO:0045944 | Positive regulation of transcription from RNA polymerase II promoter | BP | 1 × 10^-3^ |
|  | GO:1900034 | Regulation of cellular response to heat | BP | 2 × 10^-3^ |
|  | GO:0042026 | Protein refolding | BP | 2 × 10^-3^ |
|  | GO:2001240 | Negative regulation of extrinsic apoptotic signaling pathway in absence of ligand | BP | 2 × 10^-3^ |
|  | GO:0031397 | Negative regulation of protein ubiquitination | BP | 4 × 10^-3^ |
|  | GO:0019343 | Cysteine biosynthetic process via cystathionine | BP | 4 × 10^-3^ |
|  | GO:0070434 | Positive regulation of nucleotide-binding oligomerization domain containing 2 signaling pathway | BP | 4 × 10^-3^ |
|  | GO:0033209 | Tumor necrosis factor-mediated signaling pathway | BP | 5 × 10^-3^ |
|  | GO:0090084 | Negative regulation of inclusion body assembly | BP | 5 × 10^-3^ |
|  | GO:0019344 | Cysteine biosynthetic process | BP | 8 × 10^-3^ |
|  | GO:0070814 | Hydrogen sulfide biosynthetic process | BP | 8 × 10^-3^ |
|  | GO:0050852 | T cell receptor signaling pathway | BP | 1.0 × 10^-2^ |
|  | GO:0007169 | Transmembrane receptor protein tyrosine kinase signaling pathway | BP | 1.0 × 10^-2^ |
|  | GO:0019346 | Transsulfuration | BP | 1.3 × 10^-2^ |
|  | GO:0008299 | Isoprenoid biosynthetic process | BP | 1.4 × 10^-2^ |
|  | GO:0032757 | Positive regulation of interleukin-8 production | BP | 1.5 × 10^-2^ |
|  | GO:0038095 | Fc-epsilon receptor signaling pathway | BP | 1.6 × 10^-2^ |
|  | GO:0032870 | Cellular response to hormone stimulus | BP | 2.5 × 10^-2^ |
|  | GO:0090630 | Activation of GTPase activity | BP | 2.8 × 10^-2^ |
|  | GO:0007259 | JAK-STAT cascade | BP | 3.0 × 10^-2^ |
|  | GO:0045926 | Negative regulation of growth | BP | 3.2 × 10^-2^ |
|  | GO:0033173 | Calcineurin-NFAT signaling cascade | BP | 3.4 × 10^-2^ |
|  | GO:0070206 | Protein trimerization | BP | 4.2 × 10^-2^ |
|  | GO:0070098 | Chemokine-mediated signaling pathway | BP | 4.9 × 10^-2^ |
|  | GO:0000786 | Nucleosome | CC | 2.0 × 10^-2^ |
|  | GO:0031234 | Extrinsic component of cytoplasmic side of plasma membrane | CC | 3.6 × 10^-2^ |
|  | GO:0036513 | Derlin-1 retrotranslocation complex | CC | 5 × 10^-2^ |
|  | GO:0001077 | Transcriptional activator activity, RNA polymerase II core promoter proximal region sequence-specific binding | MF | 1 × 10^-4^ |
|  | GO:0000982 | Transcription factor activity, RNA polymerase II core promoter proximal region sequence-specific binding | MF | 1 × 10^-4^ |
|  | GO:0005164 | Tumor necrosis factor receptor binding | MF | 1 × 10^-3^ |
|  | GO:0055131 | C3HC4-type RING finger domain binding | MF | 1 × 10^-3^ |
|  | GO:0005524 | ATP binding | MF | 1 × 10^-3^ |
|  | GO:0051082 | Unfolded protein binding | MF | 3 × 10^-3^ |
|  | GO:0000049 | tRNA binding | MF | 3 × 10^-3^ |
|  | GO:0031072 | Heat shock protein binding | MF | 4 × 10^-3^ |
|  | GO:0000978 | RNA polymerase II core promoter proximal region sequence-specific DNA binding | MF | 8 × 10^-3^ |
|  | GO:0003677 | DNA binding | MF | 1.2 × 10^-2^ |
|  | GO:0005085 | Guanyl-nucleotide exchange factor activity | MF | 1.3 × 10^-2^ |
|  | GO:0001875 | Lipopolysaccharide receptor activity | MF | 1.3 × 10^-2^ |
|  | GO:0046872 | Metal ion binding | MF | 1.4 × 10^-2^ |
|  | GO:0005070 | SH3/SH2 adaptor activity | MF | 1.8 × 10^-2^ |
|  | GO:0003700 | Transcription factor activity, sequence-specific DNA binding | MF | 2.4 × 10^-2^ |
|  | GO:0004715 | Non-membrane spanning protein tyrosine kinase activity | MF | 2.8 × 10^-2^ |
|  | GO:0004879 | RNA polymerase II transcription factor activity, ligand-activated sequence-specific DNA binding | MF | 4.4 × 10^-2^ |
|  | GO:0001618 | Virus receptor activity | MF | 4.6 × 10^-2^ |
