# Supplementary material for: RNA sequencing analyses reveal differentially expressed genes and pathways as Notch2 targets in B-cell lymphoma
Source: Oncotarget. 2020 Dec 1;11(48):4527–40. doi: 10.18632/oncotarget.27805 (PMC7721612; doi:10.18632/oncotarget.27805)
Supplement: Supplementary file 3 [file oncotarget-11-4527-s003.docx]

**Supplementary Table 3:** List of significantly modified genes from selected GO terms with fold change ≥1 and ≤-1.

| **Significant GO Terms** | **Gene ID** | **Gene name** | **Fold change** | **Regulation** |
| --- | --- | --- | --- | --- |
| **hsa04150: mTOR signaling pathway** | ENSG00000232810 | *TNF* | 1.80 | upregulated |
|  | ENSG00000204673 | *AKT1S1* | 1.78 | upregulated |
|  | ENSG00000117461 | *PIK3R3* | 2.01 | upregulated |
|  | ENSG00000141564 | *RPTOR* | 2.41 | upregulated |
|  | ENSG00000051382 | *PIK3CB* | 1.76 | upregulated |
|  | ENSG00000121879 | *PIK3CA* | -1.63 | downregulated |
|  | ENSG00000165699 | *TSC1* | -1.62 | downregulated |
|  | ENSG00000071242 | *RPS6KA2* | 1.54 | upregulated |
| **IPR008343: Mitogen-activated protein (MAP) kinase phosphatase** | ENSG00000138166 | *DUSP5* | 2.29 | upregulated |
|  | ENSG00000158050 | *DUSP2* | 1.55 | upregulated |
|  | ENSG00000120129 | *DUSP1* | 2.21 | upregulated |
| **GO: 0045926~negative regulation of growth** | ENSG00000125144 | *MT1G* | 2.76 | upregulated |
|  | ENSG00000111653 | *ING4* | -1.51 | downregulated |
|  | ENSG00000125148 | *MT2A* | 1.66 | upregulated |
|  | ENSG00000198417 | *MT1F* | 2.30 | upregulated |
| **GO: 0007223~Wnt signaling pathway, calcium modulating pathway** | ENSG00000131196 | *NFATC1* | -1.61 | downregulated |
|  | ENSG00000138814 | *PPP3CA* | 1.70 | upregulated |
|  | ENSG00000087095 | *NLK* | -1.62 | downregulated |
|  | ENSG00000085741 | *WNT11* | 1.59 | upregulated |
|  | ENSG00000180340 | *FZD2* | -1.60 | downregulated |
| **Interleukin receptor SHC signaling** | ENSG00000100385 | *IL2RB* | 2.56 | upregulated |
|  | ENSG00000117461 | *PIK3R3* | 2.01 | upregulated |
|  | ENSG00000100368 | *CSF2RB* | 1.89 | upregulated |
|  | ENSG00000051382 | *PIK3CB* | 1.76 | upregulated |
|  | ENSG00000121879 | *PIK3CA* | -1.63 | downregulated |
| **h_tnfr2 Pathway: TNFR2 Signaling Pathway** | ENSG00000100906 | *NFKBIA* | 1.58 | upregulated |
|  | ENSG00000226979 | *LTA* | 2.70 | upregulated |
|  | ENSG00000120129 | *DUSP1* | 2.21 | upregulated |
|  | ENSG00000006062 | *MAP3K14* | 1.93 | upregulated |
|  | ENSG00000127191 | *TRAF2* | 1.88 | upregulated |
| **h_bcr Pathway: BCR Signaling Pathway** | ENSG00000131196 | *NFATC1* | -1.61 | downregulated |
|  | ENSG00000138814 | *PPP3CA* | 1.70 | upregulated |
|  | ENSG00000177606 | *JUN* | 1.66 | upregulated |
|  | ENSG00000120910 | *PPP3CC* | -1.57 | downregulated |
|  | ENSG00000101096 | *NFATC2* | 1.59 | upregulated |
|  | ENSG00000170345 | *FOS* | 3.57 | upregulated |
| **CD28 dependent PI3K/Akt signaling** | ENSG00000010810 | *FYN* | 1.70 | upregulated |
|  | ENSG00000117461 | *PIK3R3* | 2.01 | upregulated |
|  | ENSG00000101255 | *TRIB3* | 2.31 | upregulated |
|  | ENSG00000121879 | *PIK3CA* | -1.63 | downregulated |
|  | ENSG00000121594 | *CD80* | -1.55 | downregulated |
|  | ENSG00000006062 | *MAP3K14* | 1.93 | upregulated |
| **HSF1-dependent transactivation** | ENSG00000109971 | *HSPA8* | 2.17 | upregulated |
|  | ENSG00000080824 | *HSP90AA1* | 1.54 | upregulated |
|  | ENSG00000132002 | *DNAJB1* | 2.02 | upregulated |
|  | ENSG00000204673 | *AKT1S1* | 1.78 | upregulated |
|  | ENSG00000141564 | *RPTOR* | 2.41 | upregulated |
|  | ENSG00000204388 | *HSPA1B* | 2.57 | upregulated |
|  | ENSG00000204389 | *HSPA1A* | 2.58 | upregulated |
| **GO: 0031397~negative regulation of protein ubiquitination** | ENSG00000010810 | *FYN* | 1.70 | upregulated |
|  | ENSG00000130119 | *GNL3L* | 1.52 | upregulated |
|  | ENSG00000086061 | *DNAJA1* | 1.93 | upregulated |
|  | ENSG00000204388 | *HSPA1B* | 2.57 | upregulated |
|  | ENSG00000187608 | *ISG15* | 1.78 | upregulated |
|  | ENSG00000204389 | *HSPA1A* | 2.58 | upregulated |
|  | ENSG00000185238 | *PRMT3* | 1.84 | upregulated |
| **DNA Damage/Telomere Stress Induced Senescence** | ENSG00000203812 | *HIST2H2AA3* | -2.21 | downregulated |
|  | ENSG00000270882 | *HIST2H4A* | -1.54 | downregulated |
|  | ENSG00000277075 | *HIST1H2AE* | -2.16 | downregulated |
|  | ENSG00000272196 | *HIST2H2AA4* | -2.19 | downregulated |
|  | ENSG00000197238 | *HIST1H4J* | -2.22 | downregulated |
|  | ENSG00000124635 | *HIST1H2BJ* | -1.69 | downregulated |
|  | ENSG00000124762 | *CDKN1A* | 1.91 | upregulated |
|  | ENSG00000113522 | *RAD50* | 1.77 | upregulated |
| **Ca2^+^ pathway** | ENSG00000131196 | *NFATC1* | -1.61 | downregulated |
|  | ENSG00000138814 | *PPP3CA* | 1.70 | upregulated |
|  | ENSG00000087095 | *NLK* | -1.62 | downregulated |
|  | ENSG00000085741 | *WNT11* | 1.59 | upregulated |
|  | ENSG00000127588 | *GNG13* | -1.67 | downregulated |
|  | ENSG00000180340 | *FZD2* | -1.60 | downregulated |
|  | ENSG00000111664 | *GNB3* | -1.55 | downregulated |
| **VEGFA-VEGFR2 Pathway** | ENSG00000010810 | *FYN* | 1.70 | upregulated |
|  | ENSG00000027869 | *SH2D2A* | 1.59 | upregulated |
|  | ENSG00000080824 | *HSP90AA1* | 1.54 | upregulated |
|  | ENSG00000136754 | *ABI1* | -1.63 | downregulated |
|  | ENSG00000071051 | *NCK2* | 1.98 | upregulated |
|  | ENSG00000116701 | *NCF2* | 5.42 | upregulated |
|  | ENSG00000155849 | *ELMO1* | 1.67 | upregulated |
|  | ENSG00000051382 | *PIK3CB* | 1.76 | upregulated |
|  | ENSG00000121879 | *PIK3CA* | -1.63 | downregulated |
| **hsa04370: VEGF signaling pathway** | ENSG00000027869 | *SH2D2A* | 1.59 | upregulated |
|  | ENSG00000138814 | *PPP3CA* | 1.70 | upregulated |
|  | ENSG00000120910 | *PPP3CC* | -1.57 | downregulated |
|  | ENSG00000117461 | *PIK3R3* | 2.01 | upregulated |
|  | ENSG00000164867 | *NOS3* | 1.62 | upregulated |
|  | ENSG00000101096 | *NFATC2* | 1.59 | upregulated |
|  | ENSG00000051382 | *PIK3CB* | 1.76 | upregulated |
|  | ENSG00000063176 | *SPHK2* | 1.54 | upregulated |
|  | ENSG00000121879 | *PIK3CA* | -1.63 | downregulated |
| **hsa04662: B cell receptor signaling pathway** | ENSG00000131196 | *NFATC1* | -1.61 | downregulated |
|  | ENSG00000100906 | *NFKBIA* | 1.58 | upregulated |
|  | ENSG00000138814 | *PPP3CA* | 1.70 | upregulated |
|  | ENSG00000177606 | *JUN* | 1.66 | upregulated |
|  | ENSG00000120910 | *PPP3CC* | -1.57 | downregulated |
|  | ENSG00000117461 | *PIK3R3* | 2.01 | upregulated |
|  | ENSG00000101096 | *NFATC2* | 1.59 | upregulated |
|  | ENSG00000170345 | *FOS* | 3.57 | upregulated |
|  | ENSG00000051382 | *PIK3CB* | 1.76 | upregulated |
|  | ENSG00000121879 | *PIK3CA* | -1.63 | downregulated |
| **hsa04210: Apoptosis** | ENSG00000100906 | *NFKBIA* | 1.58 | upregulated |
|  | ENSG00000023445 | *BIRC3* | 1.98 | upregulated |
|  | ENSG00000169598 | *DFFB* | 1.72 | upregulated |
|  | ENSG00000173530 | *TNFRSF10D* | -1.75 | downregulated |
|  | ENSG00000117461 | *PIK3R3* | 2.01 | upregulated |
|  | ENSG00000100368 | *CSF2RB* | 1.89 | upregulated |
|  | ENSG00000051382 | *PIK3CB* | 1.76 | upregulated |
|  | ENSG00000121879 | *PIK3CA* | -1.63 | downregulated |
|  | ENSG00000006062 | *MAP3K14* | 1.93 | upregulated |
|  | ENSG00000127191 | *TRAF2* | 1.88 | upregulated |
| **GO: 0048010~vascular endothelial growth factor receptor signaling pathway** | ENSG00000010810 | *FYN* | 1.70 | upregulated |
|  | ENSG00000027869 | *SH2D2A* | 1.59 | upregulated |
|  | ENSG00000080824 | *HSP90AA1* | 1.54 | upregulated |
|  | ENSG00000136754 | *ABI1* | -1.63 | downregulated |
|  | ENSG00000071051 | *NCK2* | 1.98 | upregulated |
|  | ENSG00000116701 | *NCF2* | 5.42 | upregulated |
|  | ENSG00000204217 | *BMPR2* | 1.61 | upregulated |
|  | ENSG00000155849 | *ELMO1* | 1.67 | upregulated |
|  | ENSG00000051382 | *PIK3CB* | 1.76 | upregulated |
|  | ENSG00000121879 | *PIK3CA* | -1.63 | downregulated |
|  | ENSG00000118257 | *NRP2* | 1.75 | upregulated |
| **hsa04064: NF-kappa B signaling pathway** | ENSG00000100906 | *NFKBIA* | 1.58 | upregulated |
|  | ENSG00000065675 | *PRKCQ* | 1.50 | upregulated |
|  | ENSG00000023445 | *BIRC3* | 1.98 | upregulated |
|  | ENSG00000154589 | *LY96* | 1.89 | upregulated |
|  | ENSG00000276070 | *CCL4L2* | 3.53 | upregulated |
|  | ENSG00000136869 | *TLR4* | 1.57 | upregulated |
|  | ENSG00000082805 | *ERC1* | 1.65 | upregulated |
|  | ENSG00000226979 | *LTA* | 2.70 | upregulated |
|  | ENSG00000006062 | *MAP3K14* | 1.93 | upregulated |
|  | ENSG00000127191 | *TRAF2* | 1.88 | upregulated |
| **hsa04630: Jak-STAT signaling pathway** | ENSG00000115415 | *STAT1* | 1.64 | upregulated |
|  | ENSG00000184557 | *SOCS3* | 6.83 | upregulated |
|  | ENSG00000104432 | *IL7* | 1.87 | upregulated |
|  | ENSG00000100385 | *IL2RB* | 2.56 | upregulated |
|  | ENSG00000168610 | *STAT3* | 1.95 | upregulated |
|  | ENSG00000117461 | *PIK3R3* | 2.01 | upregulated |
|  | ENSG00000185338 | *SOCS1* | 1.59 | upregulated |
|  | ENSG00000242689 | *CNTF* | 1.67 | upregulated |
|  | ENSG00000136634 | *IL10* | 5.75 | upregulated |
|  | ENSG00000100368 | *CSF2RB* | 1.89 | upregulated |
|  | ENSG00000051382 | *PIK3CB* | 1.76 | upregulated |
|  | ENSG00000121879 | *PIK3CA* | -1.63 | downregulated |
|  | ENSG00000103522 | *IL21R* | 1.71 | upregulated |
| **IPR001452: Src homology-3 domain** | ENSG00000125089 | *SH3TC1* | 1.79 | upregulated |
|  | ENSG00000137478 | *FCHSD2* | 1.62 | upregulated |
|  | ENSG00000101336 | *HCK* | 1.95 | upregulated |
|  | ENSG00000129675 | *ARHGEF6* | 1.60 | upregulated |
|  | ENSG00000143322 | *ABL2* | 1.54 | upregulated |
|  | ENSG00000010810 | *FYN* | 1.70 | upregulated |
|  | ENSG00000004777 | *ARHGAP33* | -1.52 | downregulated |
|  | ENSG00000135605 | *TEC* | 1.60 | upregulated |
|  | ENSG00000136754 | *ABI1* | -1.63 | downregulated |
|  | ENSG00000182957 | *SPATA13* | 1.86 | upregulated |
|  | ENSG00000071051 | *NCK2* | 1.98 | upregulated |
|  | ENSG00000005379 | *TSPOAP1* | -2.02 | downregulated |
|  | ENSG00000116701 | *NCF2* | 5.42 | upregulated |
|  | ENSG00000155926 | *SLA* | 2.77 | upregulated |
|  | ENSG00000145819 | *ARHGAP26* | 1.65 | upregulated |
